# Chronic Plaque Psoriasis in Poland: Disease Severity, Prevalence of Comorbidities, and Quality of Life

**DOI:** 10.3390/jcm11051254

**Published:** 2022-02-25

**Authors:** Dorota Purzycka-Bohdan, Anna Kisielnicka, Monika Zabłotna, Bogusław Nedoszytko, Roman J. Nowicki, Adam Reich, Dominik Samotij, Justyna Szczęch, Dorota Krasowska, Joanna Bartosińska, Joanna Narbutt, Aleksandra Lesiak, Paulina Barasińska, Agnieszka Owczarczyk-Saczonek, Joanna Czerwińska, Jacek C. Szepietowski, Aleksandra Batycka-Baran, Rafał Czajkowski, Magdalena Górecka-Sokołowska, Lidia Rudnicka, Joanna Czuwara, Marta Sobalska-Kwapis, Dominik Strapagiel, Aneta Szczerkowska-Dobosz

**Affiliations:** 1 Department of Dermatology, Venereology and Allergology, Medical University of Gdansk, 80-210 Gdansk, Poland; a.kisielnicka@gumed.edu.pl (A.K.); monika.zablotna@gumed.edu.pl (M.Z.); bned@gumed.edu.pl (B.N.); rnowicki@gumed.edu.pl (R.J.N.); aneta.szczerkowska-dobosz@gumed.edu.pl (A.S.-D.); 2 Invicta Fertility and Reproductive Centre, Molecular Laboratory, 80-850 Gdansk, Poland; 3 Department of Dermatology, Institute of Medical Sciences, Medical College of Rzeszow University, 35-959 Rzeszow, Poland; adamandrzejreich@gmail.com (A.R.); dominik.samotij@gmail.com (D.S.); justyna.m.szczech@gmail.com (J.S.); 4 Department of Dermatology, Venerology and Paediatric Dermatology, Medical University of Lublin, 20-081 Lublin, Poland; dor.krasowska@gmail.com (D.K.); jbartosinski@gmail.com (J.B.); 5 Department of Dermatology, Pediatric Dermatology and Oncology, Medical University of Lodz, 90-419 Lodz, Poland; joanna.narbutt@onet.pl (J.N.); lesiak_ola@interia.pl (A.L.); paulina.barasinska@gmail.com (P.B.); 6 Department of Dermatology, Sexually Transmitted Diseases and Clinical Immunology, Collegium Medicum, University of Warmia and Mazury, 10-229 Olsztyn, Poland; agnieszka.owczarczyk@uwm.edu.pl (A.O.-S.); joannaj061@gmail.com (J.C.); 7 Department of Dermatology, Venereology and Allergology, Wroclaw Medical University, 50-367 Wroclaw, Poland; jacek.szepietowski@umed.wroc.pl (J.C.S.); aleksandra.batyckabaran@umed.wroc.pl (A.B.-B.); 8 Department of Dermatology and Venerology, Ludwik Rydygier Collegium Medicum in Bydgoszcz, Nicolaus Copernicus University in Torun, 87-100 Torun, Poland; r.czajkowski@cm.umk.pl (R.C.); magdalenagoreckaa@gmail.com (M.G.-S.); 9 Department of Dermatology, Medical University of Warsaw, 02-008 Warsaw, Poland; lidiarudnicka@gmail.com (L.R.); jczuwara@yahoo.com (J.C.); 10 Biobank Laboratory, Department of Molecular Biophysics, Faculty of Biology and Environmental Protection, University of Lodz, 90-237 Lodz, Poland; marta.sobalska@biol.uni.lodz.pl (M.S.-K.); dominik.strapagiel@biol.uni.lodz.pl (D.S.)

**Keywords:** psoriasis, epidemiology, comorbidities, environmental factors, quality of life

## Abstract

The epidemiology of psoriasis has not been widely assessed in Polish population so far. This study aimed to investigate psoriasis epidemiological situation by evaluating disease course and severity, management, comorbidities, environmental factors, and knowledge about this disorder among psoriatic patients in Poland. A cross-sectional cohort population-based study enrolled 1080 psoriatic patients and 1200 controls. The mean age of psoriasis onset was 27.6 years; 78.24% had type I psoriasis. Positive family history of psoriasis was reported in 44.81% of patients, whereas itch was reported in vast majority of patients (83.33%). Based on PASI score moderate psoriasis was the most common in studied group (mean 12.63 ± 9.33, range 0–67.2). The DLQI score (12.01 ± 7.41, range 0–30.0) indicated a very large effect of psoriasis on the quality of life. Hypertension was the most prevalent comorbidity (33.80%), followed by obesity (16.85%) and dyslipidemia (11.85%). Stress was the foremost cause of disease exacerbation (66.20%); however, infections (44.07%) and seasonal changes (45.09%) had also an impact on the course of psoriasis. Psoriatic patients were more often smokers (37.59%) vs. general population (27.50%; *p* < 0.0001). In conclusion, epidemiological studies help clinicians in better disease and patient understanding, which may translate into better management and patient compliance.

## 1. Introduction

Psoriasis is a common, chronic inflammatory disease of the skin characterized by formation of scaling plaques. Chronic plaque psoriasis constitutes the most widespread type of the disorder. The worldwide prevalence of psoriasis is estimated to reach about 0.51–11.43% of adults. The geographical and populational discrepancies of the disease incidence have been reported, with the lowest incidence observed in Africa and Asia and the highest in Scandinavian countries [1]. Such incidence variance underpins an importance of epidemiological studies of psoriasis in different parts of the globe. In small epidemiological studies among the Polish adult population, the disease incidence was estimated at 1.45–2.99% [2,3]. Moreover, psoriasis was more prevalent among population of northern Poland (Pomeranian and Kuyavian-Pomeranian voivodships) than in southern provinces (Lower Silesian) [3].

The combination of immunological, genetic, and environmental factors plays a pivotal role in psoriasis etiology [4,5]. The activation of the inflammatory immune cells and pathways in psoriasis also exerts a systemic impact on the concomitance of autoinflammatory and metabolic diseases. The most common psoriasis comorbidities involve hypertension, insulin resistance, type 2 diabetes mellitus, dyslipidemia, obesity, metabolic syndrome, and depression [6,7]. Due to various local and systemic consequences, psoriasis may lead to serious deterioration of patients’ quality of life [8]. Moreover, inhabitancy, diet, smoking, alcohol consumption, and other environmental factors play an important role in the development of skin lesions [9].

The aim of this study was to investigate the epidemiological situation of Polish psoriatic patients in respect of disease course, activity and management, as well as prevalence of comorbidities, influence of environmental factors, and knowledge about psoriasis.

## 2. Materials and Methods

### 2.1. Chronic Plaque Psoriasis Patients’ Study Group

**Figure 1:**
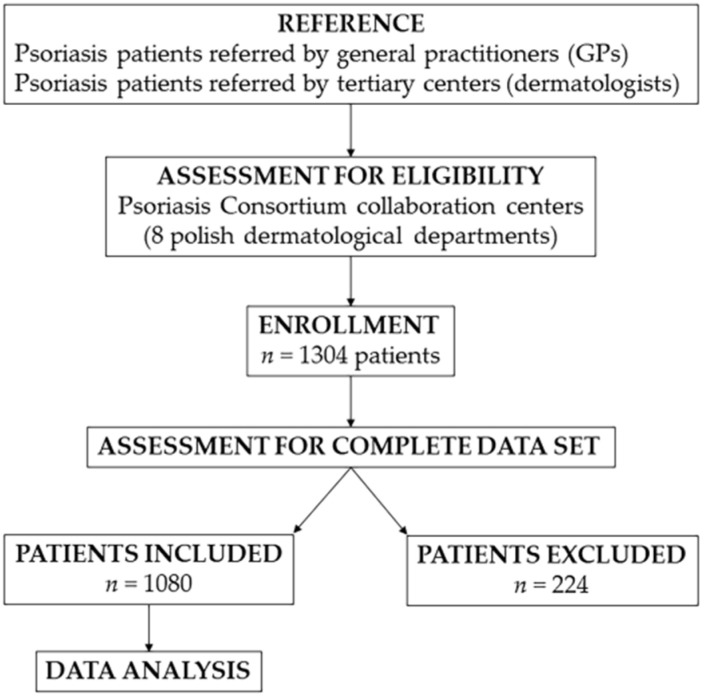
The consort diagram.

A cross-sectional cohort population-based study was carried out between 2010 and 2019 by a multicenter cooperation of 8 Polish dermatological departments called Polish Psoriasis Consortium (Figure 1).

Patients with a clinical and/or histological diagnosis of plaque psoriasis were recruited from all regions of Poland. The demographic and medical history data were collected, in particular psoriasis onset age, psoriasis clinical manifestations, factors triggering new skin lesions, itch presence and intensity in the last 24-h time period (assessed according to NRS—Numerical Rating Scale), articular afflictions and concomitant diseases (including psoriatic arthritis defined by compliance with CASPAR criteria—The Classification Criteria for Psoriatic Arthritis), family history of psoriasis, past and current psoriasis treatment, lifestyle factors (diet, alcohol, smoking), and patients’ general knowledge about the disease. Each participant underwent a medical dermatological examination with the assessment of the presence of nail involvement, psoriasis activity (size, appearance, and induration of lesions), disease severity (according to PASI—Psoriasis Area and Severity Index, BSA—Body Surface Area), impact of psoriasis on health-related life quality (measured with DLQI—Dermatological Life Quality Index), along with systolic and diastolic blood pressure range (measured by certified automatic monitors) and body mass index (BMI). Finally, samples of the peripheral blood were collected for the total cholesterol level evaluation.

The definition criteria applied in the study involved the following: type of psoriasis (I—age of onset <40.0 years old; II—age of onset ≥40.0 years old), subjective pruritus severity measured by the NRS (0–10.0) scale (>0–3.0 points—mild; 4.0–6.0 points—moderate; 7.0–10.0 points—severe) [10], psoriasis disease severity determined by PASI (<10.0 points—mild; 10.0–15.0 points—moderate; >15.0 points—severe) [11], and dermatological quality of life by use of DLQI score (0–1.0 points—no effect; 2.0–5.0 points—small effect; 6.0–10.0 points—moderate effect; 11.0–20.0 points—very large effect; 21.0–30.0 points—extremely large effect) [12].

The study was carried out in accordance with the Declaration of Helsinki, the EU directive on data protection and the standards of Good Clinical Practice. The research protocol was authorized by the Independent Bioethics Committee for Scientific Research (NKBBN/313/2017). All subjects provided written, informed consent prior to their partaking in the study.

### 2.2. Control Population

The control group (*n* = 1200) was randomly selected from the POPULOUS collection (*n* = 10,000) of the Biobank Lab of the Department of Molecular Biophysics of the University of Lodz, Poland, and registered since 2013 in the BBMRI (Biobanking and BioMolecular resources Research Infrastructure) catalog [13]. POPULOUS collection comprises of volunteers from all around Poland recruited in the years 2010–2012. The survey was conducted by a professional public opinion polling and survey-taking company (SMG/KRC Poland, a Millward Brown subsidiary) as a face-to-face interview. The non-biologically related respondents were randomly selected in a door-to-door survey. A self-report questionnaire, including demographic data, height, weight, BMI, smoking habits, and the presence of chronic underlying diseases (including psoriasis), was conducted. Participants with a clinical or histological diagnosis of chronic plaque psoriasis were excluded from the control group.

The research was approved by the University of Lodz Review Board (33/KBBN-UL/I/2018). All subjects provided written, informed consent prior to their partaking in the study. The study was carried out in accordance with ethical principles for medical investigation involving human subjects.

### 2.3. Statistical Analysis

The statistical calculations were made using Statistica v. 12.0. (StatSoft, Inc., Tulsa, OK, USA, 2015) and Microsoft Office Excel. The analysis of qualitative data was performed applying Pearson chi-square test. The independent variables satisfying the assumptions for parametric tests were analysed using Student’s *t*-test, while independent variables that did not satisfy the assumptions were analysed using non-parametric tests: Mann–Whitney U test or Kruskal–Wallis test. The correlation of qualitative data was analysed using Spearman’s rank correlation coefficient. In all tests, *p* < 0.05 was considered statistically significant.

## 3. Results

The total of 1080 psoriatic patients were enrolled in the study (mean age 46.88 ± 15.45 years, age range 8.0–93.0 years). Of those individuals, 398 (37.85%) were women (mean age 48.66 ± 16.4 years, age range 8.0–85.0 years), and 682 (63.15%) were men (mean age 45.84 ± 14.78 years, age range 15.0–93.0 years, difference between women and men *p* = 0.001). The control population encompassed 1200 individuals (mean age 43.17 ± 15.25 years, age range 20.0–77.0 years): 450 (37.50%) women (mean age 44.03 ± 15.42 years, age range 20.0–77.0 years) and 750 (62.50%) men (mean age 42.66 ± 15.13 years, age range 20.0–77.0 years, difference between women and men *p* = 0.001).

### 3.1. Chronic Plaque Psoriatic Patients’ Disease Course, Severity, and Management Evaluation

Table 1 provides the analysis of crucial chronic plaque psoriasis characteristics among Polish psoriatic patients. The results showed that mean age of the disease onset in general was 27.6 years and thus corresponds with the notable prevalence of type I psoriasis in the population of patients. Of those with type I psoriasis, 406 (48.05%) individuals had a positive family history of the disorder, whereas patients with type II psoriasis had a considerably less common positive family history of the disease (*n* = 78 (33.19%), *p* = 0.00005).

Among individuals with articular complaints, only approximately half of the subjects (53.81%) were diagnosed with psoriatic arthritis (women *n* = 93 (50.82%); men *n* = 154 (55.80%), *p* = 0.29). Altogether, a minority of chronic plaque psoriatic patients were administered disease-modifying antirheumatic drugs (DMARDs) (both csDMARDs—conventional systemic DMARDs—and bDMARDS—biologic DMARDs) (14.63%). However, a considerable number of individuals with concomitant psoriatic arthritis had a past or present history of above-mentioned treatment (*n* = 150 (60.73%), *p* < 0.0001; women *n* = 59 (63.44%); men *n* = 91 (59.10%), *p* = 0.5). 

**Figure 2:**
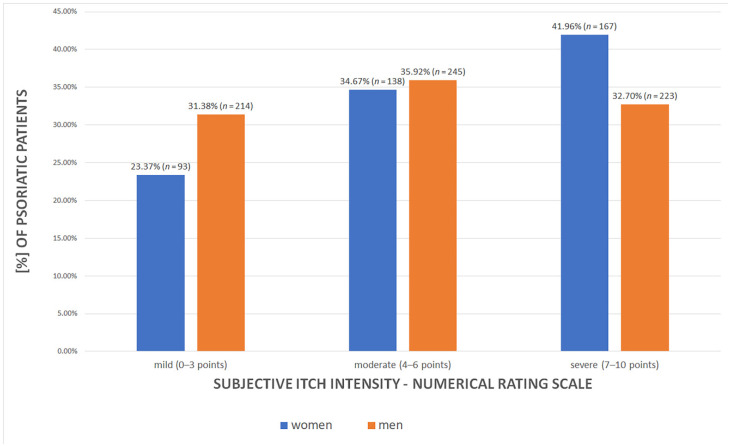
The intensity of itch in female and male psoriatic patients (*p* = 0.003).

The patients’ subjective evaluation of itch severity against mean value of the NRS scale demonstrated overall moderate level of the intensity (5.05 ± 3.03 points). Nonetheless, primarily, severe itch (*n* = 390 (36.11%), *p* = 0.003) and, secondly, moderate itch (*n* = 383 (35.46%), *p* = 0.003) intensities were reported by the patients. Figure 2 illustrates the divergence between women and men in itch sensation.

**Figure 3:**
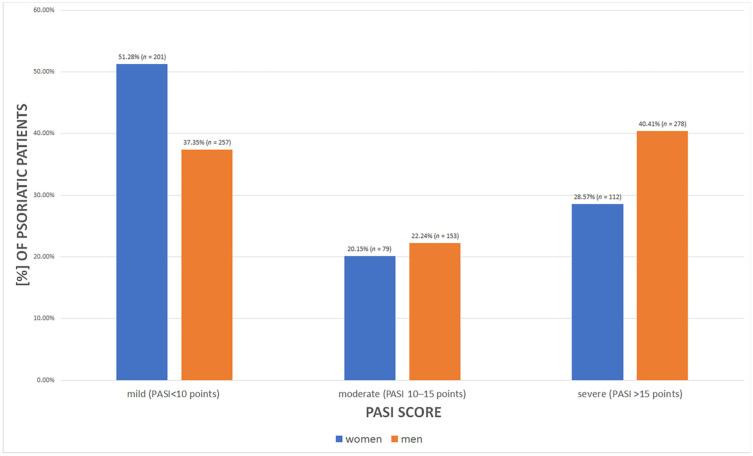
The severity of psoriasis in female and male psoriatic patients defined by PASI score (*p* = 0.00002).

The mean PASI value for both men and women indicates moderate chronic plaque psoriasis to be the most common (mean 12.63 ± 9.33 points, range 0–67.2 points). However, the analysis of the range of PASI values (<10.0 points—mild; 10.0–15.0 points—moderate; >15.0 points—severe) shows that, in fact, moderate chronic plaque psoriasis seems to be the least common (*n* = 232 (21.48%)), and mild psoriasis the most common (*n* = 458 (42.41%), *p* < 0.00001). Figure 3 depicts a greater occurrence of the mild form of psoriasis among women, whereas men tend to develop mild and severe psoriasis.

The mean DLQI score (12.01 ± 7.41 points, range 0.0–30.0 points) for chronic plaque psoriatic patients indicates a very large effect on health-related quality of life deterioration. Furthermore, the analysis of the results showed a positive correlation (R = 0.56, *p* < 0.001) between the disease severity (PASI) and patients’ life quality (DLQI).

### 3.2. Psoriasis and Comorbid Diseases

**Figure 4:**
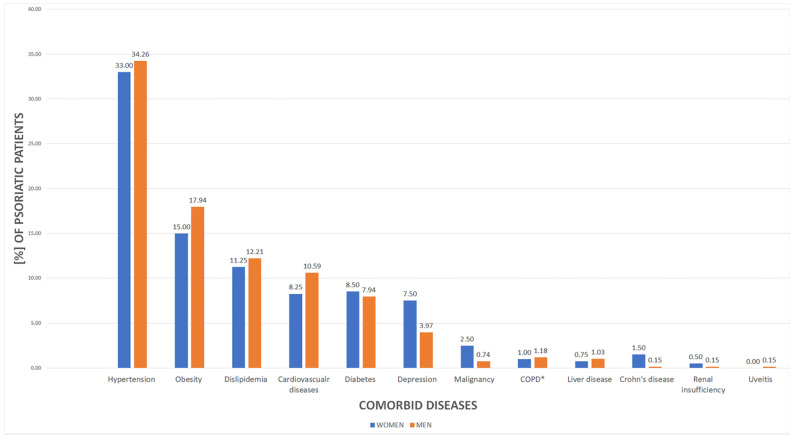
The prevalence of comorbid diseases among female and male psoriatic patients. * COPD, chronic obstructive pulmonary disease.

Approximately half of psoriatic patients, women (*n* = 203 (50.75%)) and men (*n* = 342 (50.29%), *p* = 0.88), respectively, reported to have comorbid diseases. Among them, hypertension was the most prevalent (*n* = 365 (33.80%), *p* < 0.0001), followed by obesity (*n* = 182 (16.85%), *p* < 0.0001) and dyslipidemia (*n* = 128 (11.85%), *p* < 0.0001). Figure 4 illustrates data correlations in female and male psoriatic patients.

Table 2 provides the analysis of medical and laboratory parameters of the main concomitant disorders among psoriatic patients.

**Figure 5:**
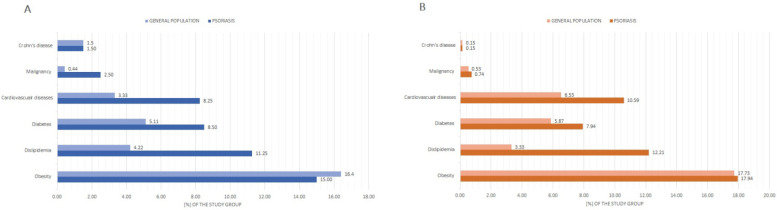
The comparison of concomitant diseases among psoriasis patients and general population. (**A**) Female psoriasis patients vs. female control group. (**B**) Male psoriasis patients vs. male control group.

Furthermore, in comparison to general population (referenced as control group), both female and male psoriatic patients were reported to have a greater prevalence of dyslipidemia, diabetes, cardiovascular disorders, and malignancies (Figure 5).

### 3.3. Psoriasis and the Environmental Factors

**Figure 6:**
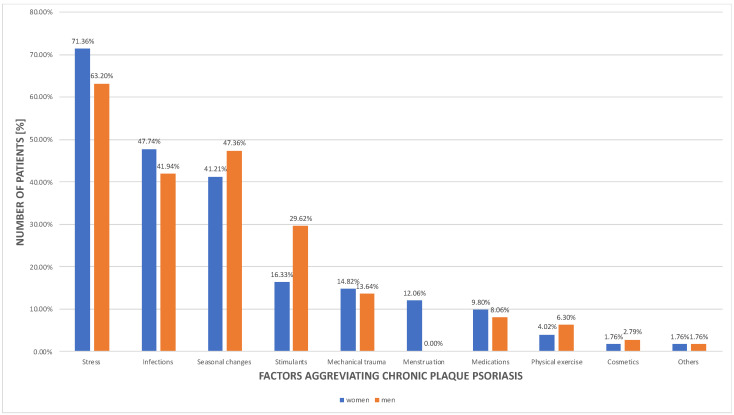
Patients’ subjective assessment of the factors aggravating the psoriasis disease course.

Figure 6 demonstrates psoriatic patients’ subjective assessment of the environmental factors aggravating skin lesions with distribution among sexes. Stress was the most important cause of chronic plaque psoriasis exacerbation (*n* = 715 (66.20%)). Secondly, infectious diseases (*n* = 476 (44.07%)) and seasonal changes (*n* = 487 (45.09%)) were reported to have a considerable impact on the psoriasis disease course.

As far as the inhabitancy conditions are concerned, the proportion of both psoriatic patients (*n* = 679 (62.87%)) and the control group (*n* = 727 (60.58%), difference between the patients and controls *p* = 0.26) living in highly urbanized areas was comparable. There was a significant predominance of psoriatic patients claiming to abstain from excessive alcohol consumption, both among women (*n* = 387 (97.24%)) and men (*n* = 619 (90.76%), *p* = 0.00005). Likewise, the majority of psoriatic patients negated being on a special diet due to psoriatic burden (women *n* = 345 (86.68%); men *n* = 610 (89.44%), *p* = 0.17).

In general, psoriatic patients were more often active smokers (*n* = 406 (37.59%)) in comparison to the general population (*n* = 330 (27.50%), difference between patients and controls *p* < 0.0001). On the contrary, among non-psoriatic individuals, there was a greater number of past smokers (*n* = 353 (29.42%)) in comparison to psoriatic patients (*n* = 163 (15.09%), difference between the controls and patients *p* < 0.0001). Table 3 presents smoking status among women and men in psoriasis and control group.

### 3.4. Knowledge about Psoriasis

The psoriatic patients’ self-assessment of their knowledge of the disease revealed that over a half of them (*n* = 537 (63.25%)) considered to have a good understanding of the disorder. They also believed that healthy individuals in general do not possess adequate knowledge about psoriasis and ranked it as bad (*n* = 545 (64.27%)).

**Figure 7:**
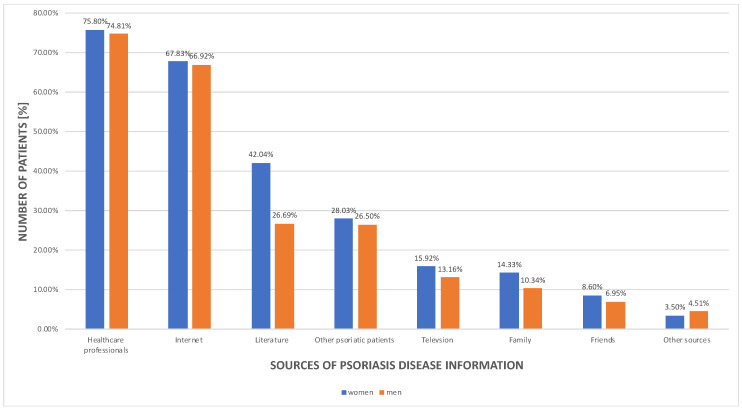
Analysis of the psoriatic patients’ choice of the disease information source.

The psoriasis knowledge sources are presented in Figure 7 for women and men, respectively.

## 4. Discussion

To our knowledge, this is the first epidemiological study of psoriasis among Polish population on the national scale in the context of disease severity, prevalence of comorbidities, and social burden.

### 4.1. Disease Course, Activity, and Management Evaluation

Our study reveals that type I of chronic plaque psoriasis dominates among Polish patients and constitutes about 78% of cases. Moreover, it is correlated with higher prevalence of positive family history of the disease and thus greater genetic predisposition. On the contrary, type II of chronic plaque psoriasis was significantly less common and did not correspond with notable family history trait. These findings are consistent with previous local (Szczerkowska-Dobosz A. et al. in Poland) and worldwide studies conducted in both Asian (Chen L. et al.) and European populations (Queiro R. et al.) [14,15,16]. 

Furthermore, nail involvement is a very common disease feature in Polish psoriatic patients (63.61% as reported in our study). Therefore, it is important for clinicians in general to perform a thorough skin examination at each medical visit, with extraordinary attention being paid to the specific body locations, such as nails, scalp, face, and genital area. The analysis of the national cross-sectional study (PsoHealth2) performed in Germany showed a 35.6% prevalence of nail psoriasis [17]. The Danish Skin Cohort evaluation of psoriasis epidemiology reported that nails were affected in 24.5% of psoriatic patients [18]. Interestingly, smaller nail psoriasis prevalence (16.0%) was reported in the observational study of the Corrona Psoriasis Registry (The United States of America) [19]. Due to the fact that nail psoriasis causes major psychological and functional burden [18], very often requires prolonged and complex treatment [20], and is associated with greater psoriatic arthritis susceptibility [21], early screening for nail alterations and therapy might be beneficial for patients.

Our study revealed an unsettling phenomenon of a considerable number of articular complaints (almost half of the study group) among polish psoriatic patients. The investigation of the National Health Fund (NHF) of psoriatic arthritis (PsA) epidemiology in Poland pinpointed increasing prevalence of the disorder in the recent decade (nowadays 73.11 cases per 100,000) [22,23]. Alinaghi’s et al. meta-analysis showed that those data are relatable to populations of European ancestry suffering from psoriasis (PsA prevalence equal to 22.7%) [24]. Therefore, our findings may suggest the still-timely problem of the underdiagnosis of psoriatic arthritis in Polish patients. With joint pain being the primary PsA symptom, more caution and screening are necessary in clinical practice in order to reduce severe disease outcome and disabilities. However, it is worth considering other common causes of articular pains, such as degenerative joint disease and gout, which also may influence the misinterpretation of PsA epidemiology.

Recently, the problem of itch among psoriatic patients is a field of robust investigation. Current knowledge about itch pathology in psoriasis suggests complex interplay between neuropeptides, cytokines and interleukins (expressly IL-31), hormones, and the vascular system [25]. According to various global researchers, there is an evidence for growing number of itch sensation among psoriatic patients. Sampogna F. et al. reported that 63.8% of psoriatic patients hospitalized in Italy experienced itch sensation [26]. Interestingly, Stinco G. et al. observed even up to 80.0% itch prevalence among Italian patients [27]. Analysis by Mrowietz U. et al. of the PRISTINE study showed that even 96.0% of patients with moderate and severe psoriasis suffered from itch, and 62.0% of them had a severe form of itch [28]. Correspondingly, independent local studies in the Polish population (by Czarnecka-Operacz M. et al., Reich A. et al. and Szepietowski J.C. et al.) reported very high frequency of itch sensation in psoriatic patients, ranging from 60.0% to 90.0% [29,30,31]. The data are coherent with our analysis, which states that the considerable number of psoriatic patients (more than 80.0%) suffers from itch. Moreover, women were more likely to report higher frequency and greater intensity of itch sensation than men. Some studies also described such correlation; however, the exact pathomechanism of greater susceptibility is yet to be determined [26,32,33]. Although various analyses confirm that itch leads to major deterioration of patients’ quality of life [34,35], it seems that the issue still deserves attention [36] and should urge clinicians to seek itch-oriented care and treatment.

With regard to psoriasis management in Polish patients, biologic therapy is still the least common therapeutic option. Although in recent times, there has been immense progress in the development of new treatment targets, biological therapies still have major drawbacks, such as high cost and availability limited only to experienced dermatological centers [37]. Rencz F. et al. analyzed management of biological psoriasis therapies in Central and Eastern European countries (including Poland) and found that only about 0.25% of patients were administered biologics. However, the authors concluded that therapy with hindered availability is more related to eligibility criteria rather than pharmacoeconomic issues [38]. Since then, major alterations in the Polish health national program (NFZ—Narodowy Fundusz Zdrowia, Warsaw, Poland) have been enforced, which have enabled greater number of patients to meet the therapeutic conditions [39]. Despite this, some principal psoriasis treatment modalities, such as apremilast and dimethyl fumarate, are not currently reimbursed in Poland; hence, there are no data considering their use [40]. Greater access to the validated psoriasis treatment options in the future might contribute to better disease management among Polish patients. Moreover, we observed that the use of oral retinoids was more prevalent in men than in women. This phenomenon may result from the teratogenic adverse effect of the treatment, which implies a long period of oral contraception and therefore constitutes a major drawback of this therapy in young women [41].

### 4.2. Comorbidities

Psoriasis is associated with greater prevalence of cardiovascular and metabolic comorbidities [42,43] and the resulting greater morbidity [44]. Our study showed that among Polish psoriatic patients, hypertension, obesity, and dyslipidemia constitute the most widespread group of concomitant diseases. Furthermore, diabetes, dyslipidemia, and cardiovascular diseases were more frequent among psoriatic patients in comparison to the control group, which represented general population of Poland. Surprisingly, the difference between the study groups in the context of obesity prevalence was not significant. This could be explained by the fact that in the recent decade, there has been a continuous increase in overweight and obesity prevalence among he Polish population in general (30.0–40.0% of population is overweight and 10.0–20.0% obese) [45,46]. The latest epidemiological analyses of the PURE Poland cohort study reported an even greater increase in obesity frequency in Polish population, reaching about 30.0% of the population [47]. What is worth emphasizing, the genetic association of obesity is sex-dependent in Polish population [48]. Correspondingly, Mallbris L. et al. reported the obesity prevalence among Swedish psoriatic patients to be 10.0% and was insignificantly less frequent than in control group (13.0%) [49].

According to our results, hypertension is a primary comorbid disease among psoriatic patients regardless of gender (about 33.0% of patients). The NATPOL study, to date the greatest epidemiological database of cardio-metabolic diseases in Polish population, described the prevalence of hypertension in general population to reach about 30.0% [50]. Therefore, the differences between our findings and general population are minor. However, Armstrong A.W. et al. provided a systematic review and a meta-analysis comprising 309,469 psoriatic patients, which showed that the odds ratio for the hypertension among them was 1.58 in comparison to the control population [51].

Early screening and prevention of comorbidities among psoriatic patients may contribute to the cardiovascular events-risk reduction and thus morbidity and better life quality.

### 4.3. Social and Lifestyle Burden

In psoriatic patients’ self-observation, stress constitutes the most common disease-aggravating factor. According to other researchers, stress was proven to be responsible for the exacerbation or recurrence of psoriatic lesions in about 70.0% of patients of European origin [52,53]. Besides, it leads to earlier onset [54] and greater severity of the disease [55]. It is worth mentioning that there is also evidence that psoriasis has a substantial impact on the greater risk of depression and suicidal behavior [56]. For these reasons, psychological counselling is an important issue for psoriatic patients. Treatment should concentrate not only on skin but also on stress management and mental health. Among other commonly reported psoriasis-aggravating factors were infections, seasonal changes, drugs, mechanical trauma, obesity, and diet, which is consistent with our investigation [57,58,59]. Moreover, especially in the COVID-19 pandemic era, disease exacerbation is notable due to the co-existence of multiple factors, such as stress, infection, and less active lifestyle [60]. Patients’ education about triggering factors and their avoidance is important, as it may enforce better patient–dermatologist cooperation and raise treatment compliance. 

We evaluated that psoriatic patients, men in particular, tended to be more frequently active smokers in comparison to the general population. The meta-analysis of prevalence studies confirmed the relationship between psoriasis and about 1.7 times greater frequency of smoking [61]. Moreover, smoking is a well-known disease-aggravating factor, poor treatment-outcome predictor, and cardiovascular event-risk factor [62,63]. Therefore, it is surprising that despite evident negative impact of smoking on psoriasis, patients still continued smoking. It is possible that stress due to lowered life quality may contribute to the phenomenon of self-driving into bad habits. 

On the other hand, less than 10.0% of Polish psoriatic patients declared excessive alcohol consumption. These data seem to show significant discrepancies between other studies. Brenaut et al. performed an extensive systematic literature review, which concluded that alcohol consumption prevalence among psoriatic patients was greater than in general population [64]. Furthermore, consistent with WHO (World Health Organization, Geneva, Switzerland) report on alcohol consumption in Poland (2016), the total alcohol drinking per capita reaches almost 12.0 L of pure alcohol per year and is slightly higher than in other European countries [65]. Hence, we speculate that, in fact, our results could be underestimated due to bias in self-report on patients’ alcohol habits resulting from the shame to admit alcohol abuse.

The study revealed a very interesting trend in patients’ assessment of their level of psoriasis knowledge. It seems that psoriatic patients seek information about their health status; however, at the same time the social awareness of the disorder is not sufficient enough. Reich et al. demonstrated that about 10.0–20.0% of psoriatic patients in south Poland shared unrealistic beliefs about the disease course and management [66]. Not only psoriatic patients but also society in general should be educated about the disease since false beliefs of its infectious etiology are still present. Good knowledge and understanding of the disease may help patients with the disease management, decrease social burden, and boost patients’ life quality. Healthcare professionals should focus on good patient education because they are the main source of information. Moreover, medical services should focus on reliable online information sources, as patients very often refer to them.

### 4.4. Limitations of the Study

The study has several limitations. Firstly, the POPULOUS database lacked data concerning hypertension and alcohol consumption. However, we were able to compare our results in the discussion section with other reliable data registries, such as NATPOL (hypertension) and WHO (alcohol consumption) records. Secondly, we did not evaluate the distinctive features of psoriasis in pediatric population. Thirdly, due to the multi-center design of the study, there could be bias regarding subjective interpretation of data, including patients’ skin examination and patient-reported information.

## 5. Conclusions

Despite the limitations of the study, we present noteworthy results concerning epidemiological situation of psoriasis patients in Poland. Our study highlights the importance of nail involvement, joint pain, or pruritus among psoriatic patients, which should be assessed in daily practice due to its high prevalence and health consequences. Furthermore, early screening and prevention of comorbidities among psoriatic patients, particularly hypertension and obesity, may contribute to the reduction of cardiovascular risk and thus morbidity and better life quality. Finally, the study emphasizes the need for public education about psoriasis and highlights limitations associated with the disease. To conclude, epidemiological studies on psoriasis may help clinicians in better disease and patient understanding, which translates into better disorder management and patient compliance.

## Figures and Tables

**Table 1:** Disease characteristics of the Polish chronic plaque psoriasis patients.

Variable	General *n* = 1080	Women *n* = 398	Men *n* = 682	*p*-Value (Difference between Women and Men)
**Mean age (years ± SD)**	46.9 ± 15.4	48.7 ± 16.4	45.8 ± 14.8	0.001
**Mean age of chronic plaque psoriasis onset (years ± SD)**	27.6 ± 15.4	27.8 ± 17.3	27.5 ± 14.2	0.15
**Type of chronic plaque psoriasis (%)**				
I	845 (78.24%)	296 (74.37%)	549 (80.50%)	0.02
II	235 (21.76%)	102 (25.63%)	133 (19.50%)	
**Family history for psoriasis (%)**				
Positive	484 (44.81%)	182 (45.73%)	302 (44.28%)	0.64
**Nail involvement (%)**				
Present	687 (63.61%)	230 (57.79%)	457 (67.01%)	0.002
**Articular afflictions (%)**				
Present	459 (42.50%)	183 (45.98%)	276 (40.47%)	0.08
**Psoriatic arthritis diagnosis (%)**				
Present	254 (23.52%)	93 (23.37%)	161 (23.61%)	0.93
**Current rheumatological treatment (%)**				
Present	158 (14.63%)	61 (15.33%)	97 (14.22%)	0.62
**Itch (%)**				
Present	900 (83.33%)	337 (84.67%)	563 (82.55%)	0.37
**Subjective itch intensity (%)**				
10-point NRS mean value (points ± SD)	5.05 ± 3.03	5.48 ± 3.09	4.80 ± 2.97	0.0001
**Psoriasis activity (%)**				
Continuous formation of new lesions (active)	851 (78.80%)	302 (75.88%)	549 (80.50%)	0.07
**Skin lesions morphology (%)**				
Lesions size <3 cm	302 (27.96%)	120 (30.15%)	182 (26.69%)	0.16
Lesions size >3 cm	765 (70.83%)	269 (67.59%)	496 (72.73%)	
**Mean PASI (points ± SD)**	12.63 ± 9.33	10.81 ± 8.5	13.67 ± 9.62	<0.00001
**Mean BSA (% ± SD)**	22.52 ± 20.22	18.87 ± 17.85	24.65 ± 21.21	<0.00001
**Mean DLQI (points ± SD)**	12.01 ± 7.41	12.34 ± 7.56	11.83 ± 7.32	0.27
**Disease severity (PASI) and life quality (DLQI) correlation *n* = 1065**	R = 0.56 (Spearman’s rank correlation), *p* < 0.001			
**Current and past chronic plaque psoriasis treatment (%)**				
Topical	1029 (95.28%)	390 (96.06%)	639 (94.81%)	0.35
Phototherapy (UVB, PUVA)	642 (59.40%)	229 (56.40%)	413 (61.28%)	0.11
Oral methotrexate	576 (53.33%)	209 (51.48%)	367 (54.45%)	0.34
Oral cyclosporine	345 (31.94%)	125 (30.79%)	220 (32.64%)	0.53
Systemic retinoids	269 (24.91%)	81 (19.95%)	188 (27.89%)	0.003
Biological therapy	247 (22.87%)	91 (22.41%)	156 (23.15%)	0.78

**Table 2:** The characteristics of the most prevalent comorbid diseases in psoriatic patients.

Variable	General *n* = 1080	Women *n* = 398	Men *n* = 682	*p*-Value (Difference between Women and Men)
**Hypertension**				
Mean systolic blood pressure (mmHg ± SD)	133.3 ± 17.01	128.62 ± 15.53	135.95 ± 17.25	<0.00001
Mean diastolic blood pressure (mmHg ± SD)	80.58 ± 10.17	77.82 ± 9.47	82.15 ± 10.23	<0.00001
**Obesity**				
Mean BMI (kg/m^2^ ± SD)	27.83 ± 5.35	27.32 ± 5.75	28.13 ± 5.07	0.01
**Dyslipidemia**				
Total cholesterol level (mg/dl ± SD)	183.41 ± 46.05	186.41 ± 43.45	181.76 ± 47.39	0.13

**Table 3:** Smoking status in females and males in the patients’ and control groups.

Smoking Status	Females			Males		
	Psoriatic Patients’ Group *n* = 398	Control Group *n* = 450	*p*-Value (Difference between Women in Psoriasis and Control Group)	Psoriatic Patients’ Group *n* = 682	Control Group *n* = 750	*p*-Value (Difference between Men in Psoriasis and Control Group)
**Current smokers (%)**	123 (30.90%)	105 (23.33%)	0.01	283 (41.50%)	225 (30.00%)	<0.0001
**Past smokers (%)**	42 (10.55%)	108 (24.00%)	<0.0001	121 (17.74%)	245 (32.67%)	<0.0001
**Non-smokers (%)**	233 (58.54%)	237 (52.67%)	0.09	278 (40.76%)	280 (37.33%)	0.18

## Data Availability

The data that support the findings of this study are available from the corresponding author upon reasonable request.
